# Clinical Characteristics and Management of Pediatric Facial Wounds with Documented Infection or Delayed Presentation: A 25-Year Retrospective Case Series

**DOI:** 10.3390/jcm15197479

**Published:** 2026-09-25

**Authors:** Manuela Jaindl, Vanessa Groß, Lara Marie Bogensperger, Antonia Schwarz, Britta Chocholka, Stephan Payr

**Affiliations:** 1 University Clinic of Orthopedics and Trauma Surgery, Department of Trauma Surgery, Medical University of Vienna, 1090 Vienna, Austria; manuela.jaindl@meduniwien.ac.at (M.J.); n11916964@students.meduniwien.ac.at (V.G.); lara.bogensperger@meduniwien.ac.at (L.M.B.); n12220006@students.meduniwien.ac.at (A.S.); britta.chocholka@meduniwien.ac.at (B.C.); 2 Section of Pediatric Trauma Surgery, Department of Trauma Surgery, University Clinic of Orthopedics and Trauma Surgery, Medical University of Vienna, 1090 Vienna, Austria

**Keywords:** pediatric facial wounds, wound infection, delayed presentation, retrospective case series, antibiotic use

## Abstract

**Background/Objectives:** Evidence on infected and delayed pediatric facial wounds outside the setting of animal bites is limited. This study characterized the anatomy, wound characteristics, management, and documented outcomes of pediatric facial wounds with documented infection or delayed/non-recent presentation. **Methods:** We retrospectively reviewed patients younger than 18 years, identified by electronic free-text search, who presented at a single tertiary trauma center from 2000 to 2024. The hospital recorded 24,877 pediatric facial-wound presentations during the same period. Records were classified as clinically infected when the source dataset coded infection as present and as delayed/non-recent when infection was coded absent but the record described delayed or non-recent presentation. Descriptive analyses and exploratory two-sided Fisher exact tests used variable-specific denominators. **Results:** Of 92 screened records, 89 met the inclusion criteria: 61 were record-coded as clinically infected and 28 as delayed/non-recent. The selected cohort represented 0.36% of all pediatric facial-wound presentations. Perioral wounds accounted for 46/89 cases and forehead/periorbital wounds for 34/89. Wounds were superficial in 36/89 (40%), subcutaneous in 35/89 (39%), intramuscular in 15/89 (17%), and of unknown depth in 3/89 (3%). Initial closure was documented in 22/89 (25%). All eight dog-bite wounds occurred in the infected group, and none had been initially closed. Post-presentation antibiotics were documented in 48/58 (83%) evaluable infected and 5/28 (18%) delayed/non-recent records; admission was documented in 26/59 (44%) and 2/28 (7%), respectively. **Conclusions:** This case series describes a small, selected subset of pediatric facial-wound presentations. Mechanism, depth, anatomy, and management differed between the two record-defined groups, but the retrospective sampling and classification framework precludes causal or incidence inference.

## 1. Introduction

Pediatric facial wounds are common because the head and face are frequently involved in childhood trauma [1,2,3,4]. Most facial lacerations heal well, but infection, periorbital cellulitis, scarring, and other complications can require additional treatment [5,6,7,8,9,10]. Published descriptions consistently identify the perioral, periorbital, nasal, and frontal regions as common sites [3,11,12,13,14,15]. However, site-specific infection risk is difficult to infer because most cohorts do not combine a denominator-defined population with detailed information on the mechanism, depth, timing, and initial wound management.

Evidence supporting antibiotic prophylaxis for traumatic facial wounds remains heterogeneous. Contemporary recommendations emphasize the wound mechanism, tissue damage, contamination, bite exposure, patient factors, and established clinical infection rather than anatomical site alone [16,17,18,19]. Pediatric evidence is concentrated on animal bites [20,21,22,23,24,25,26], whereas infected or delayed facial wounds from other mechanisms are less well characterized. Host wound-healing responses and facial skin microbial communities vary with age and context [27,28]. Emerging biomaterial research has expanded wound-dressing functions from passive coverage to antimicrobial delivery, sensing, and healing assessment, while experimental immunoregulatory hydrogels have targeted oxygenation and reactive oxygen species in chronic wounds [29,30]. These technologies are not directly validated for acute pediatric facial wounds, but they underscore the importance of precise wound characterization when interpreting clinical management.

The objective of this study was to characterize the anatomy, mechanism, wound type, size and depth, initial closure, antibiotic treatment, microbiology, follow-up, and documented complications of pediatric facial wounds with record-documented infection or delayed/non-recent presentation. We additionally described dog bites and oral/intraoral wounds separately and explored selected associations with record-defined group membership.

## 2. Materials and Methods

This retrospective, single-center case series study was conducted at the Department of Trauma Surgery, Medical University of Vienna. The study period extended from 1 January 2000 through 31 December 2024. The Ethics Committee of the Medical University of Vienna approved the study (1133/2025) in accordance with the Declaration of Helsinki.

Potentially eligible records were identified in the hospital electronic information system (AKIM) by free-text searching combinations of concepts for facial location, wound type, infection or inflammation, and delayed or non-recent presentation. A separate broad administrative count identified 24,877 pediatric facial-wound presentations among patients aged 0–17 years during 2000–2024. The targeted free-text search was not validated against this denominator, and its sensitivity may have varied over time. All retrieved records were manually reviewed. Inclusion required age younger than 18 years, a facial soft-tissue wound, and classification into one of the two mutually exclusive source-record groups shown in Table 1. Records were excluded when the wound was outside the facial region, neither operational group was supported by the documentation, or information was insufficient for classification.

The source spreadsheet contained 92 screened patient records. Three lacked sufficient information for operational classification, leaving 89 patients for analysis; each patient contributed one encounter and one wound record. The variables included age, sex, injury type, bite mechanism, documented infection status, facial region, wound size and depth, calendar-day interval to presentation, pre-presentation antibiotics, initial wound closure and closure method, antibiotic treatment after presentation, hospital admission, surgery, microbiological testing, follow-up, and complications. Wound size was documented as length in mm and wound depth was re-abstracted from the narrative clinical documentation and categorized according to the deepest documented tissue layer involved: superficial, when the wound was limited to the skin/dermis without documented subcutaneous extension; subcutaneous, when extension into subcutaneous tissue was documented without muscle involvement; intramuscular, when the wound extended into or through muscle; and unknown, when the available documentation was insufficient to assign one of these categories. Perioral records were re-reviewed to distinguish external, intraoral, and combined wounds; vermilion involvement; and intraoral self-bite versus laceration. The source comorbidity field combined chronic conditions, allergies, and trauma-associated diagnoses and was excluded. Macroscopic contamination and foreign-body status were excluded because ascertainment was incomplete. Anatomical coding was reconciled with diagnosis text: one nonstandard code with an explicit buccal diagnosis was classified as buccal, and one combined oral/buccal entry was retained as a combined perioral category.

### Statistical Analysis

Categorical variables are presented as n/N (%) using the number of records with an interpretable value as the denominator; missing or unclear values were not recoded as no. Percentages were rounded to whole numbers because of the small cohort, except the cohort’s proportion of all facial-wound presentations, which was retained to two decimal places to avoid rounding to zero. Continuous variables are summarized by their median and interquartile range (IQR), with the range where informative. Frontal and orbital wounds were combined as forehead/periorbital, while oral, buccal, and combined oral/buccal wounds were combined as perioral for aggregate analyses. Exploratory associations between record-defined infection status and region, bite mechanism, wound depth, initial closure, admission, and post-presentation antibiotics were assessed with two-sided Fisher exact tests. Odds ratios (ORs) and 95% confidence intervals (CIs) were calculated using the standard logit method; tests were unadjusted for multiple comparisons and are hypothesis-generating. Multivariable regression was not performed because of sparse cells, quasi-separation, and the different record-based group definitions. No formal sample-size calculation was performed; all eligible records identified during the 25-year period were included. A post hoc descriptive sensitivity analysis divided the period into 2000–2007, 2008–2015, and 2016–2024. Analyses were performed using Microsoft Excel (version 16.90.2) and independently checked against the patient-level spreadsheet. OpenAI ChatGPT (GPT-5; accessed 23. August 2026) assisted with code drafting for data verification. All classifications and calculations were independently verified by the authors.

## 3. Results

### 3.1. Study Population and Cohort Characteristics

The 89 included patients comprised 58 boys (65%) and 31 girls (35%). Their median age was 4 years (IQR, 2–8; range, 1–17). The source record coded 61 patients (69%) as clinically infected and 28 (31%) as delayed/non-recent without record-coded infection. These 89 selected cases represented 0.36% of the 24,877 pediatric facial-wound presentations recorded during the same period. This proportion describes cohort selection and should not be interpreted as infection incidence because the targeted search was not validated against the denominator. No standardized chronic-comorbidity variable was available.

Lacerations were the most frequent injury type (63/89, 71%), followed by bites (20/89, 22%), stab wounds (4/89, 4%), one cut, and one excoriation. The bite wounds comprised eight dog bites (9%) and 12 human bites (13%).

Perioral wounds were the most frequent regional category (46/89, 52%): 29 oral, 16 buccal, and one combined oral/buccal wound. Forehead/periorbital wounds accounted for 34/89 (38%): 18 frontal and 16 orbital. The remaining locations were chin (n = 5), auricular (n = 2), nasal (n = 1), and other (n = 1).

### 3.2. Management, Follow-Up, and Missingness

The median calendar-day interval from injury to presentation was 2 days (IQR, 1–3; range, 0–90). Follow-up was scheduled or required in 41/87 evaluable records (47%); two records were unclear. Admission was documented in 28/87 (32%), surgery in 8/88 (9%), microbiological testing in 10/88 (11%), pre-presentation antibiotics in 5/88 (6%), and post-presentation antibiotics in 53/86 (62%) (Table 2). Among the 53 records with post-presentation treatment, a regimen containing amoxicillin–clavulanate was documented in 44, another regimen was documented in four, and the agent was unavailable in five. The route was recoverable in 34/53 records: combined intravenous and oral therapy in 23, oral therapy alone in nine, and intravenous therapy alone in two; the route was unclear in 19. The duration and treatment indication were not consistently recoverable. Five complications were documented among 84 evaluable records (6%); five had unclear status.

### 3.3. Record-Coded Clinically Infected Wounds

The record-coded clinically infected group included 61 patients. Their median age was 4 years (IQR, 2–9; range, 1–17); 38 (62%) were boys and 23 (38%) were girls.

The injury types were laceration (42/61, 69%), dog bite (8/61, 13%), human bite (8/61, 13%), and stab wound (3/61, 5%).

Forehead/periorbital wounds accounted for 27/61 (44%; frontal n = 16, orbital n = 11) and perioral wounds for 27/61 (44%; oral n = 13, buccal n = 14). The other locations were chin (n = 5), auricular (n = 1), and other (n = 1).

Among the evaluable records, admission was documented in 26/59 (44%), surgery in 8/60 (13%), and microbiological testing in 9/60 (15%).

Pre-presentation antibiotic therapy was documented in 5/60 (8%) evaluable records and post-presentation treatment in 48/58 (83%). A regimen containing amoxicillin–clavulanate was documented in 41/48 treated records.

Five complications were documented among 56 infected-group records with interpretable status (9%); status was unclear in five. Four involved forehead/periorbital wounds and one involved the chin. The small number of events precluded a reliable complication model.

### 3.4. Delayed/Non-Recent Wounds Without Record-Coded Infection

The delayed/non-recent group included 28 patients. Their median age was 4 years (IQR, 2–7.25; range, 1–17); 20 (71%) were boys and eight (29%) were girls.

The injury types were laceration (21/28, 75%), human bite (4/28, 14%), stab wound (1/28, 4%), cut (1/28, 4%), and excoriation (1/28, 4%). No dog bite was present in this group.

Perioral wounds accounted for 19/28 (68%; oral n = 16, buccal n = 2, combined oral/buccal n = 1) and forehead/periorbital wounds for 7/28 (25%; frontal n = 2, orbital n = 5). One auricular and one nasal wound completed the distribution.

Admission was documented in 2/28 records (7%); no surgery was documented. Complication status was interpretable in all 28 records, and no complication was documented.

No pre-presentation antibiotic use was documented. Post-presentation antibiotics were documented in 5/28 records (18%), including a regimen containing amoxicillin–clavulanate in three.

Microbiological testing was documented in 1/28 delayed/non-recent records (4%) and in 10/88 evaluable records overall (11%). Wound swabbing was documented in all 10 tested records, with additional bacteriology coding in five. Organisms, available resistance profiles, and recorded antibiotic concordance are presented in Supplementary Table S1.

### 3.5. Group-Wise Descriptive Findings

The operational groups differed in terms of injury mix, anatomy, and management (Table 3). Boys accounted for 62% of the infected group and 71% of the delayed/non-recent group. Bites accounted for 26% and 14%, respectively; all eight dog bites were in the infected group. Table 3 summarizes injury, anatomical, and management variables within each operational group.

Forehead/periorbital wounds comprised 44% of infected-group records and 25% of delayed/non-recent records. Perioral wounds comprised 44% and 68%, respectively.

Post-presentation antibiotics and hospital admission were documented more frequently in the infected group. Variable-specific denominators account for missing values.

### 3.6. Expanded Wound Characterization and Exploratory Findings

The wound depth was superficial in 36/89 records (40%), subcutaneous in 35/89 (39%), intramuscular in 15/89 (17%), and unknown in 3/89 (3%). Among records with known depth, subcutaneous or intramuscular wounds occurred in 43/59 infected-group records (73%) and 7/27 delayed/non-recent records (26%). Initial closure was documented in 22/89 records (25%): 13 sutures, six Steri-Strips, two other techniques, and one tissue adhesive. The closure frequency was 16/61 (26%) in the infected group and 6/28 (21%) in the delayed/non-recent group.

Among the 46 perioral wounds, external involvement was documented in 22/40 evaluable records (55%), intraoral involvement in 17/40 (43%), and both in 1/40 (3%); six were not classifiable. Vermilion involvement was documented in 12/46 (26%). Among 34 perioral records with a classifiable intraoral mechanism, 20 (59%) were self-bite wounds and 14 (41%) were lacerations. These variables distinguish oral mucosal injury from external lip or buccal injury rather than treating all perioral wounds as biologically equivalent.

In exploratory two-sided Fisher exact tests, infected-group membership was associated with subcutaneous/intramuscular versus superficial depth (OR 7.68, 95% CI 2.73–21.61; *p* < 0.001), admission (OR 10.24, 95% CI 2.22–47.17; *p* < 0.001), and post-presentation antibiotics (OR 22.08, 95% CI 6.76–72.07; *p* < 0.001). Perioral location was less frequent in the infected group (OR 0.38, 95% CI 0.15–0.96; *p* = 0.043). Associations with forehead/periorbital location, any bite mechanism, dog bite, and initial closure were not statistically significant (Table 4). These unadjusted analyses describe record-defined group membership and do not establish infection risk or treatment effects.

The median wound size was 10 mm (IQR, 5–15) in both groups; several larger wounds in the infected group accounted for its wider range.

### 3.7. Dog-Bite and Perioral Subgroup Findings

All eight dog-bite wounds were in the record-coded infected group. Six were buccal and two were coded as other/nonstandard facial sites. One was superficial, three were subcutaneous, and four were intramuscular. None had been initially closed. Pre-presentation antibiotic status was evaluable in seven dog-bite records (one yes and six no); post-presentation treatment was documented in 5/7, admission in 4/7, surgery in 1/7, and microbiological testing in 1/7. Complication status was evaluable and negative in 7/7; one transferred patient had unavailable subsequent outcome data.

Among the 12 human-bite wounds, three had been initially closed (two with Steri-Strips and one with sutures); none received pre-presentation antibiotics. Because all dog-bite wounds belonged to the infected group and none had been initially closed, this dataset could not be used to compare infection outcomes after immediate versus delayed dog-bite closure.

### 3.8. Exploratory Exact Analyses

Table 4 presents the prespecified exploratory comparisons requested during peer review. Effect estimates refer to odds of membership in the record-coded infected group rather than population infection risk.

### 3.9. Post Hoc Sensitivity Analysis by Calendar Era

Case composition and management varied across the study period. Records coded as infected accounted for 29/30 (97%) in 2000–2007, 15/34 (44%) in 2008–2015, and 17/25 (68%) in 2016–2024. Post-presentation antibiotic treatment, admission, and surgery also varied (Table 5). Surgery declined from 6/29 (21%) in 2000–2007 to 2/34 (6%) in 2008–2015 and 0/25 in 2016–2024. These post hoc findings are descriptive and were not tested inferentially.

## 4. Discussion

This case series describes 89 selected pediatric facial-wound records with documented clinical infection or delayed/non-recent presentation, representing 0.36% of the 24,877 pediatric facial-wound presentations recorded at the center during the same period. The expanded analysis distinguishes dog from human bites, external from intraoral wounds, and superficial from subcutaneous or intramuscular wounds. The perioral region was the most common wound site, and management intensity was greater in the infected group.

Forehead/periorbital and perioral wounds showed different distributions across the operational groups. The exploratory analyses suggested lower odds of infected-group membership for perioral wounds and higher odds, although imprecisely estimated, for forehead/periorbital wounds. These findings cannot be interpreted as site-specific infection risk because the denominator by anatomical site and a representative noninfected comparison cohort were unavailable.

The median wound size and IQR were identical in both groups (10 mm; IQR, 5–15), although several larger wounds in the infected group widened its range. This pattern indicates substantial overlap in wound size between groups, whereas subcutaneous or intramuscular wounds were more frequent in the infected group. Depth is clinically relevant to tissue damage and contamination, but it was retrospectively abstracted from narrative documentation rather than measured using a standardized protocol. The observed association may therefore reflect documentation and referral severity as well as biology.

Post-presentation antibiotics and admission were strongly associated with infected-group membership, as expected when clinicians intensify treatment for established infection. These associations do not show prophylactic efficacy and are vulnerable to confounding by indication. The agent and route could be partly reconstructed, but the duration, treatment indication, and timing relative to closure were inconsistent. Initial closure was not associated with group membership, and none of the eight dog-bite wounds had been initially closed; a comparison of immediate versus delayed closure after dog bite was therefore not possible.

Microbiological testing was selective and documented in only 10/88 evaluable records. The small, clinician-selected subset and incomplete susceptibility data preclude pathogen prevalence estimates or conclusions about comparative antibiotic effectiveness.

The post hoc era analysis revealed substantial temporal heterogeneity, including a decline in surgery from 21% in 2000–2007 to 0% in 2016–2024. This could reflect changing case mix or severity, evolving referral thresholds, greater ambulatory management, antibiotic stewardship, different surgeons and services, and changes in electronic documentation and search sensitivity. Surgeon identifiers were not consistently available, and the small number of operations across many clinicians precluded surgeon-level outcome analysis. Calendar-era findings should therefore not be interpreted as evidence that one period’s management was superior.

Additional limitations include the retrospective single-center design, unvalidated search strategy, nonstandardized classification, variable-specific missing data, and incomplete or inconsistently recorded follow-up. The wound depth and initial treatment were reconstructed from clinical notes and were not recorded using uniform prospective definitions. The surgeon’s identity, exact closure timing, debridement details, treatment indication, route, and antibiotic duration were incomplete or unavailable. The source comorbidity field did not reliably distinguish chronic conditions from allergies and trauma-associated diagnoses, and contamination and foreign-body status were incompletely ascertained. The exploratory tests were unadjusted, involved correlated variables and sparse cells, and could not support causal inference.

This study complements the predominantly dog-bite-focused literature by describing a selected complicated subset across multiple mechanisms and by adding wound depth, initial closure, perioral anatomy, microbiology, and calendar-era management [20,21,22,23,24,25,26]. Its contribution is a detailed description of actual conditions over 25 years and the identification of variables that future studies should capture consistently.

Future prospective multicenter studies should enroll all pediatric facial wounds; apply standardized infection, anatomical, depth, and closure definitions; prospectively record the surgeon/service, timing, debridement details, and antibiotic indication, route and duration; and ensure a sufficient sample size for adjusted analyses stratified by wound mechanism.

## 5. Conclusions

In this retrospective case series, 89 selected infected or delayed/non-recent records represented 0.36% of 24,877 pediatric facial-wound presentations. The wound mechanism, depth, anatomy, and management differed between the record-defined groups. Because of targeted retrospective ascertainment, heterogeneous documentation, and confounding by indication, the findings characterize a complicated clinical subset but do not estimate infection incidence or treatment effectiveness. Prospective multicenter studies with standardized definitions are needed to evaluate site-specific infection risk and treatment outcomes.

## Figures and Tables

**Table 1:** Operational record-based classification used in the analysis.

Operational Group	Source-Record Rule	Interpretive Boundary
Record-coded clinically infected wound	The source-dataset infection indicator was coded yes, supported by clinician diagnosis or documentation of infection/inflammation.	Delay was not required, and no uniform prospective infection-severity scale was available.
Delayed/non-recent wound without record-coded infection	The source-dataset infection indicator was coded no and the clinical record described delayed or non-recent presentation.	Calendar dates did not contain time-of-day; a strict >24 h threshold could therefore not be verified retrospectively.

**Table 2:** Overall management, follow-up, and documented complications.

Parameter	Status	n	%	N
Surgery	No	80	91	88
	Yes	8	9	88
Hospital admission	No	59	68	87
	Yes	28	32	87
Microbiological testing	No	78	89	88
	Yes	10	11	88
Antibiotics before presentation	No	83	94	88
	Yes	5	6	88
Antibiotics after presentation	No	33	38	86
	Yes	53	62	86
Initial wound closure	No	67	75	89
	Yes	22	25	89
Complication	No	79	94	84
	Yes	5	6	84
Follow-up scheduled/required	No	46	53	87
	Yes	41	47	87

Note: N is the evaluable denominator. The numbers of missing/unclear values were: surgery 1, admission 2, microbiology 1, antibiotics before presentation 1, antibiotics after presentation 3, complication status 5, and follow-up status 2. Percentages may not total 100 because of rounding.

**Table 3:** Descriptive comparison of the two operational record-based groups.

Characteristic	Infected n/Summary	%	N	Delayed n/Summary	%	N
Total	61	69	89	28	31	89
Age, years, median (IQR)	4 (2–9)		61	4 (2–7.25)		28
Sex						
Female	23	38	61	8	29	28
Male	38	62	61	20	71	28
Injury type						
Laceration	42	69	61	21	75	28
Dog bite	8	13	61	0	0	28
Human bite	8	13	61	4	14	28
Stab wound	3	5	61	1	4	28
Cut	0	0	61	1	4	28
Excoriation	0	0	61	1	4	28
Facial region						
Frontal	16	26	61	2	7	28
Orbital	11	18	61	5	18	28
Forehead/periorbital	27	44	61	7	25	28
Oral	13	21	61	16	57	28
Buccal	14	23	61	2	7	28
Combined oral/buccal	0	0	61	1	4	28
Perioral	27	44	61	19	68	28
Nasal	0	0	61	1	4	28
Chin	5	8	61	0	0	28
Auricular	1	2	61	1	4	28
Other	1	2	61	0	0	28
Wound depth						
Superficial	16	26	61	20	71	28
Subcutaneous	28	46	61	7	25	28
Intramuscular	15	25	61	0	0	28
Unknown	2	3	61	1	4	28
Initial wound management						
Initial closure: yes	16	26	61	6	21	28
Steri-Strips	3	19	16	3	50	6
Suture	10	63	16	3	50	6
Other technique	2	13	16	0	0	6
Tissue adhesive	1	6	16	0	0	6
Perioral subgroup						
External	15	68	22	7	39	18
Intraoral	6	27	22	11	61	18
External and intraoral	1	5	22	0	0	18
Vermilion involved	7	26	27	5	26	19
Intraoral self-bite	11	58	19	9	60	15
Intraoral laceration	8	42	19	6	40	15
Management/outcomes						
Hospital admission: yes	26	44	59	2	7	28
Surgery: yes	8	13	60	0	0	28
Microbiology: yes	9	15	60	1	4	28
Antibiotics before presentation: yes	5	8	60	0	0	28
Antibiotics after presentation: yes	48	83	58	5	18	28
Complication: yes	5	9	56	0	0	28

Note: Basic anatomical subregions are mutually exclusive. Aggregate forehead/periorbital and perioral categories were derived from these subregions. For perioral anatomy, a variable-specific denominator was used after re-review. Closure-method percentages used the number initially closed as the denominator. N denotes the evaluable denominator; percentages may not total 100 because of rounding.

**Table 4:** Exploratory associations with record-coded infected-group membership.

Characteristic	Infected Yes/N (%)	Delayed Yes/N (%)	OR (95% CI)	Fisher *p*
Forehead/periorbital	27/61 (44)	7/28 (25)	2.38 (0.88–6.43)	0.103
Perioral	27/61 (44)	19/28 (68)	0.38 (0.15–0.96)	0.043
Any bite	16/61 (26)	4/28 (14)	2.13 (0.64–7.10)	0.279
Dog bite	8/61 (13)	0/28 (0)	Not estimable	0.053
Subcutaneous/intramuscular depth	43/59 (73)	7/27 (26)	7.68 (2.73–21.61)	<0.001
Initial closure	16/61 (26)	6/28 (21)	1.30 (0.45–3.79)	0.793
Hospital admission	26/59 (44)	2/28 (7)	10.24 (2.22–47.17)	<0.001
Antibiotics after presentation	48/58 (83)	5/28 (18)	22.08 (6.76–72.07)	<0.001

Note: ORs compare odds of record-coded infected-group membership. Depth comparison excludes three unknown-depth records. ORs use standard logit 95% CIs; *p* values are from two-sided Fisher exact tests. Tests were unadjusted and exploratory.

**Table 5:** Post hoc descriptive sensitivity analysis by calendar era.

Era	n	Infected n (%)	Delayed n (%)	Antibiotics After Presentation Yes/N (%)	Admission Yes/N (%)	Surgery Yes/N (%)
2000–2007	30	29 (97)	1 (3)	23/29 (79)	12/29 (41)	6/29 (21)
2008–2015	34	15 (44)	19 (56)	14/34 (41)	8/34 (24)	2/34 (6)
2016–2024	25	17 (68)	8 (32)	16/23 (70)	8/24 (33)	0/25 (0)

Note: N varies where source values were missing or unclear. Calendar eras were examined post hoc and are descriptive only. Percentages may not total 100 because of rounding.

## Data Availability

The datasets generated and/or analyzed in the current study are not publicly available due to data privacy but are available from the corresponding author on reasonable request.

## Supplementary Materials

The following supporting information can be downloaded at https://www.mdpi.com/article/10.3390/jcm15197479/s1, Table S1: Microbiological testing, organisms, available resistance profiles, and documented antibiotic selection in the 10 tested records.
