# Supplementary material for: Clinical Characteristics and Management of Pediatric Facial Wounds with Documented Infection or Delayed Presentation: A 25-Year Retrospective Case Series
Source: J Clin Med. 2026 Sep 25;15(19):7479. doi: 10.3390/jcm15197479 (PMC13666419; doi:10.3390/jcm15197479)
Supplement: Supplementary file 1 [file jcm-15-07479-s001.zip › jcm-4532232-supplementary.pdf]

Supplementary Table S1

Microbiological testing, organisms, available resistance profiles, and documented antibiotic selection in the 10 tested records

Cases are de-identified and ordered for presentation only. 'Concordance' reproduces the assessment recorded in the supplemental source sheet; it is not an independent guideline-based adjudication.

Case	Group	Region	Mechanism	Specimen coding	Organism(s)	Resistance/profile and recorded concordance	Documented antibiotic regimen
M1	Infected	Orbital	Human bite	Wound swab	Viridans streptococci; H. parainfluenzae; commensal Neisseria spp.; C. albicans	Profile available; listed linezolid, vancomycin, cefotaxime, or ampicillin; non-concordant	Amoxicillin–clavulanate
M2	Infected	Buccal	Dog bite	Wound swab + bacteriology	Pasteurella multocida	Profile available; listed imipenem, ceftazidime, and levofloxacin; concordant	Intravenous then oral amoxicillin–clavulanate
M3	Infected	Buccal	Laceration	Wound swab + bacteriology	No cells or microorganisms detected	Not applicable	Intravenous then oral amoxicillin–clavulanate
M4	Infected	Chin	Laceration	Wound swab + bacteriology	MRSA; beta-hemolytic streptococci	Profile available; listed erythromycin, clindamycin, or vancomycin; non-concordant	Amoxicillin–clavulanate in supplemental source; route unavailable
M5	Infected	Chin	Laceration	Wound swab	Beta-hemolytic streptococci; S. aureus	Profile available; listed doxycycline; non-concordant	Intravenous ampicillin–sulbactam followed by oral amoxicillin–clavulanate
M6	Infected	Frontal	Laceration	Wound swab	Aeromonas sobria	Profile unavailable	Not documented
M7	Infected	Frontal	Laceration	Wound swab + bacteriology	Group A beta-hemolytic streptococci	Profile unavailable	Intravenous amoxicillin–clavulanate followed by oral clindamycin
M8	Infected	Frontal	Laceration	Wound swab	Organism result not documented	Profile unavailable	Intravenous and oral clindamycin
M9	Infected	Frontal	Laceration	Wound swab + bacteriology	S. aureus in main sheet; supplemental resistance sheet coded no organism	Profile unavailable	Intravenous and oral amoxicillin–clavulanate; prior oral therapy also documented
M10	Delayed/non-recent	Orbital	Laceration	Wound swab	Group A beta-hemolytic streptococci	Profile available; listed penicillin G, moxifloxacin, erythromycin, clindamycin, or doxycycline; concordant	Intravenous ampicillin–sulbactam

Abbreviations: M, microbiology case; spp., species. Microbiological testing was performed in 10/88 evaluable records (11%). Resistance/susceptibility information was available in five records. Selective testing and incomplete profiles precluded pathogen prevalence estimates and comparative conclusions about antibiotic effectiveness.
